# Clinical implementation of the Versius robotic surgical system in visceral surgery-A single centre experience and review of the first 175 patients

**DOI:** 10.1007/s00464-022-09526-x

**Published:** 2022-08-24

**Authors:** Stefan Wehrmann, Kristin Tischendorf, Torsten Mehlhorn, Annelie Lorenz, Michael Gündel, Hagen Rudolph, Lutz Mirow

**Affiliations:** Departement of General and Visceral Surgery, Klinikum Chemnitz gGmbH, Medical Campus of the Technische Universität Dresden, Chemnitz, Germany

**Keywords:** Robotic surgery, Versius, Minimal invasive surgery, Robotic system

## Abstract

**Background:**

Robotic surgical systems introduce new opportunities for the minimal accessed surgeon. The combination of three-dimensional magnified vision and articulated instruments with seven degrees of freedom provide a good and safe alternative to laparoscopic surgery. Indeed some of these features may support the case that robotic surgery may be better than conventional surgery. In this study, we report our experience of robotic surgery by using the first open console, modular robotic platform in Germany, the Versius Surgical System®.

**Methods:**

We implemented the Versius Surgical System® in April 2021 at our centre. Since then, 175 patients received robotic assisted surgery. All patients were included in this study. Data were analysed by using the SPSS (IBM Statistics) Software.

**Results:**

175 patients underwent robotic surgery. We started the implementation of the system by performing cholecystectomy. After the first 50 successful operations, we began to perform robotic assisted oncological resections. We saw a learning curve with improvements in total operative time and console time until reaching a standard similar to conventional laparoscopic surgery. The perioperative complication-ratio was equivalent for operations matched the histopathological outcome (MERCURY graduation, R0-staus) at oncological resections. However, four patients had to be revised because of secondary bleeding. Interestingly the total hospital stay for right sided hemicolectomy and oesophagus-resection was shorter than in laparoscopic surgery.

**Summary:**

In our opinion, the Versius Surgical System® seems to be a good, promising system and a safe alternative to other robotic systems, although any comparison is still missing. The open design enabling a better communication between console surgeon and bedside-unit assistant as well as the mobile bedside units are very interesting and allow more flexibility. Nevertheless, there are limitations of the system that require a direct comparison with other robotic systems as well as continuous advancement.

During the last three decades, minimal access surgery (MAS) has become established in all fields of abdominal and thoracic surgery. Reduced postoperative pain, lower blood loss and a shorter hospital stay are the most important advantages over conventional open surgery [1]. However, MAS requires the acquisition of laparoscopic skills that are difficult to acquire. The learning curve to reach a high standard is long. Two-dimensional imaging, limitation of movement, action tremor and even camera controlling seem to be pitfalls in learning laparoscopic surgery.

Robotic surgery helps to overcome some of these challenges. Three-dimensional imaging, tremor filtration, a much higher optical magnification and an independent operator-navigated camera are explicit advantages over laparoscopic surgery. The addition of wristed instruments allowing seven degrees of freedom further simplify the technical aspects of surgery. Furthermore, reduction of physical stress and minimisation of muscle fatigue for the surgeon is an added benefit of robotic surgery. [2–4].

The Versius Surgical System® is a new tele-operated surgical robotic system intended for use of robotic assisted surgery [5]. The system comprises the open surgeon console with hand controllers to manipulate the arms and camera of the four bedside units (BSU), which can be placed flexibly at the operating table. Each BSU supports an instrument or the endoscopic camera. The surgeon receives three-dimensional high definition video-feedback from the camera via head- up display. The open design facilitates better communication between bedside team and surgeon as well as a high flexibility in port-placement following the surgeon to replicate as far as possible the setup of conventional surgery. This also allows a seamless change to laparoscopy if needed [5, 6]. Versius was developed to mimic the articulation of the human arm and wrist. Altogether, the instruments provide seven degrees of freedom at the instrument tip allowing a much better and more specific surgical approach than laparoscopic surgery [7]. Versius does not need a specialized infrastructure (e.g. special operating theatres). Because of its modular system, each instrument and visualization arm is mobile and can be moved by its own- wheeled cart between operating rooms. Furthermore, this allows the opportunity of hybrid-techniques (e.g. robotic-laparoscopic/robotic-hand-assisted) [5]. The operating team consists of the lead surgeon performing the operating steps at the surgeon console and the bedside assistant, manipulating the robotic arms and realizing additional manual tasks as instructed by the lead surgeon.

We implemented the Versius Surgical System® in April 2021 at our centre. Since then, 175 patients underwent surgical treatment by using robotic surgery. Hence, we wish to report the short-term outcomes of our patients who have undergone robot-assisted abdominal operations.

## Material and methods

### Patients

The present retrospective study includes 175 patients who received robotic surgery by using the CMR Versius Surgical Robot System in our hospital between 1st April 2021 and 24th March 2022 (excepting the Covid-19 lockdown in Germany from October 2021 to January 2022). All patients underwent physical examination, Hemogram and biochemistry, and appropriate imaging using sonography, contrast-enhanced computed tomography or magnetic resonance tomography scan before operation. Informed consent for surgical procedure and robotic register study was obtained from each patient before robotic surgery was conducted.

To get a sufficient assessment of the usability of the robotic system, our patient selection was liberal. Patients with BMI between 17 kg/m^2^ and 47 kg/m^2^ received robotic surgery across an age range of 18 and 87 years. For the first operations we selected patients with an American Society of Anaesthesiologists (ASA) Score I or II. After the first 50 successfully treated cases, we expanded until ASA III.

The Institutional Review Board (IRB) determined that IRB approval was not required for this study as no identifying patient information was used. All patient data were collected and analysed by using SPSS.

### Surgical team and surgical procedure

Respectively two surgical teams for visceral surgery consisting of the lead surgeon and the BSU assistant as well as BSU- staff were established. Herefore, a special online training has to be successfully completed in which the technical background as well as the theoretical basics like arm position and docking, use of the optical or coagulating devices etc. Next step of schooling is a training program included in the console. Here 12 levels have to be completed to learn the handling of the console, without using the instrument-arms. After this, the whole team completed the 3.5 day Versius training programme [8] before starting the first operation. Hereby, special instructors from CMR train the surgeon and assistant while operating cadaver- firstly animal than human. Individual port positions are carved out to make the surgical procedure as comfortable as possible for patient and surgeon.

**Fig. 1:**
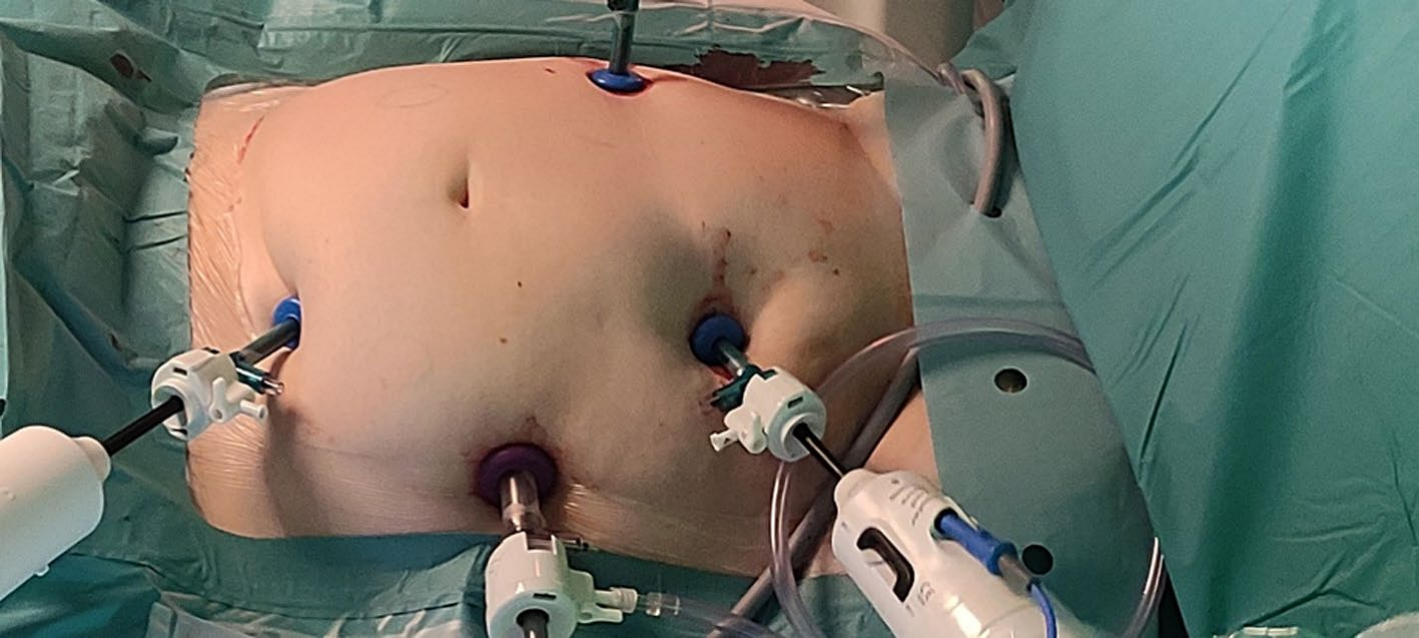
Port placement for right sided colectomy

**Fig. 2:**
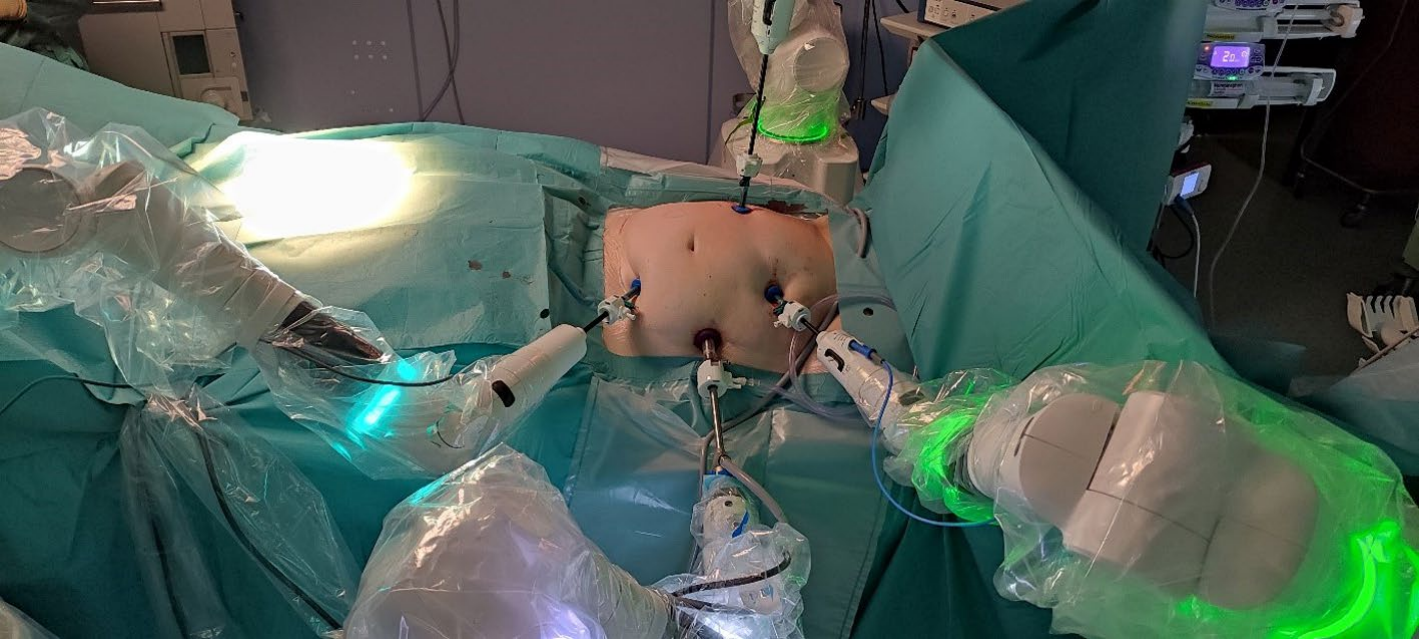
Intraoperative situs of right sided colectomy

Even the role of the BSU assistant has to be trained here. Steps like moving the instrumental arms during operation or vessel clipping in agreement with the console surgeon and, if needed a fast switch to laparoscopy are trained. Furthermore, we intermediately trained by using the console training program hereafter before starting the first surgical procedure on living human. We started with cholecystectomy to become familiar with the system. Port positions (Figs. 1 and 2) for cholecystectomy were used taken from the relevant traditional laparoscopic procedures. Major resections like pancreas or oesophagus were performed as hybrid interventions with a combination of robotic and laparoscopic/open approach. Therefore, we performed the main part of surgical preparation robotically, with some of the additional steps carried out laparoscopically.

### Surgical approach

130 cholecystectomies were performed at our hospital. Port position was harmonized to the conditions of robotic surgery. After video-laparoscopy, the fundus of the gallbladder is clamped to explore cystic duct and artery. After identification of both structures, the BSU-assistant ligates them by using Hem-o-lock clips. Hereafter, the gallbladder is released from the liver. We retrieve the gallbladder through the median port access.

Major resections like colon-resection, pancreas or oesophagus were performed as hybrid- interventions, robotic and laparoscopically/open. Therefore, we performed the main part of surgical preparation robotically. When this part was finished and further steps were needed (e.g. low anterior resection), this part was prepared laparoscopically or open.

For right sided colectomy we used port position shown in Fig. 1. After docking the bedside units (Fig. 2), the lateral approach was used with mobilization of the right sided colon robotically. After identification of the ileocolic artery, the BSU-assistant ligated it using Hem-o-lock clips. Following that, the right sided colon is dissected culminating in a complete mesocolic excision. After identification and ligation of the right colic artery, the sample was retrieved through a right lateral incision. The entero-anastomosis was sutured by hand.

**Fig. 3:**
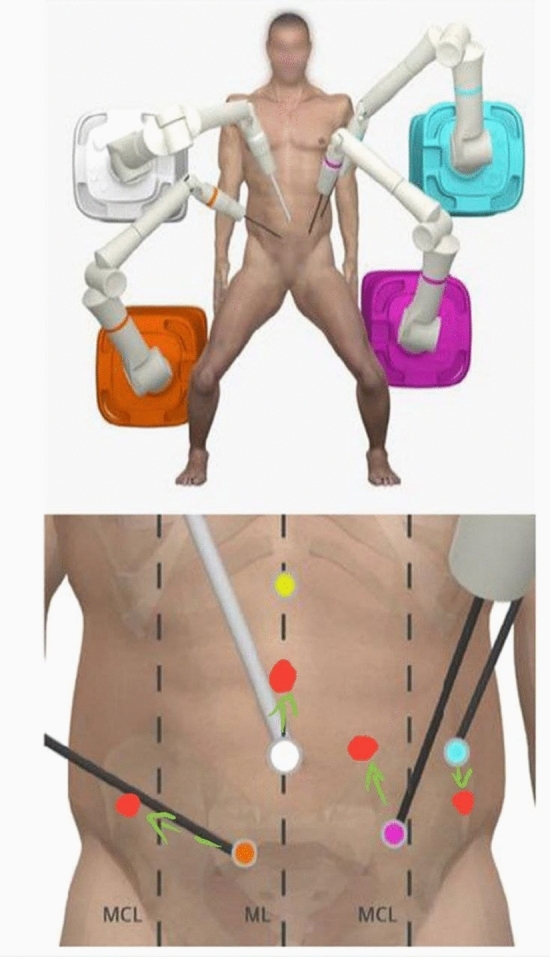
Planning of the port positions for low anterior resection

For low anterior resection (Fig. 3), the procedure started with mobilization of the splenic flexure and the descending colon on Gerota’s fascia laparoscopically. Hereafter, robotic instruments were placed. The sigmoid colon and rectum as well as the total mesorectal excision was performed robotically. In consideration of the fact, that a robotic stapler is not available yet, the rectal dissection was performed laparoscopically. The specimen was retrieved by mini-laparotomy. Finally, the anastomosis is achieved by using a circular-stapler (*Ethicon endoscopic curved intraluminal stapler*).

In the distal oesophageal resection with gastro-oesophageal anastomosis, the abdominal steps are performed robotically. After port placement, the Lesser sac is opened allowing skeletonization of the greater curvature of the stomach beyond the gastroepiploic vessels. A similar approach is taken at the lesser curvature with preservation of the right gastric artery. After D2 lymphadenectomy, the V.coronaria ventriculi and the left gastric artery are ligated using Hem-o-lock clips. The oesophageal hiatus is then opened. To prepare the sleeve, the fundus is dissected over the lesser curvature with the help of an endoscopic stapler. The thoracic part is completed by conventional laparoscopy or open surgery. For anastomosis, a circular stapler is used.

### Documentation

All patient data were collected pre-operative as well as operation time and post-operative outcome. We analysed the data by using SPSS.

## Results

### Patients’ data and oncological results

**Fig. 4:**
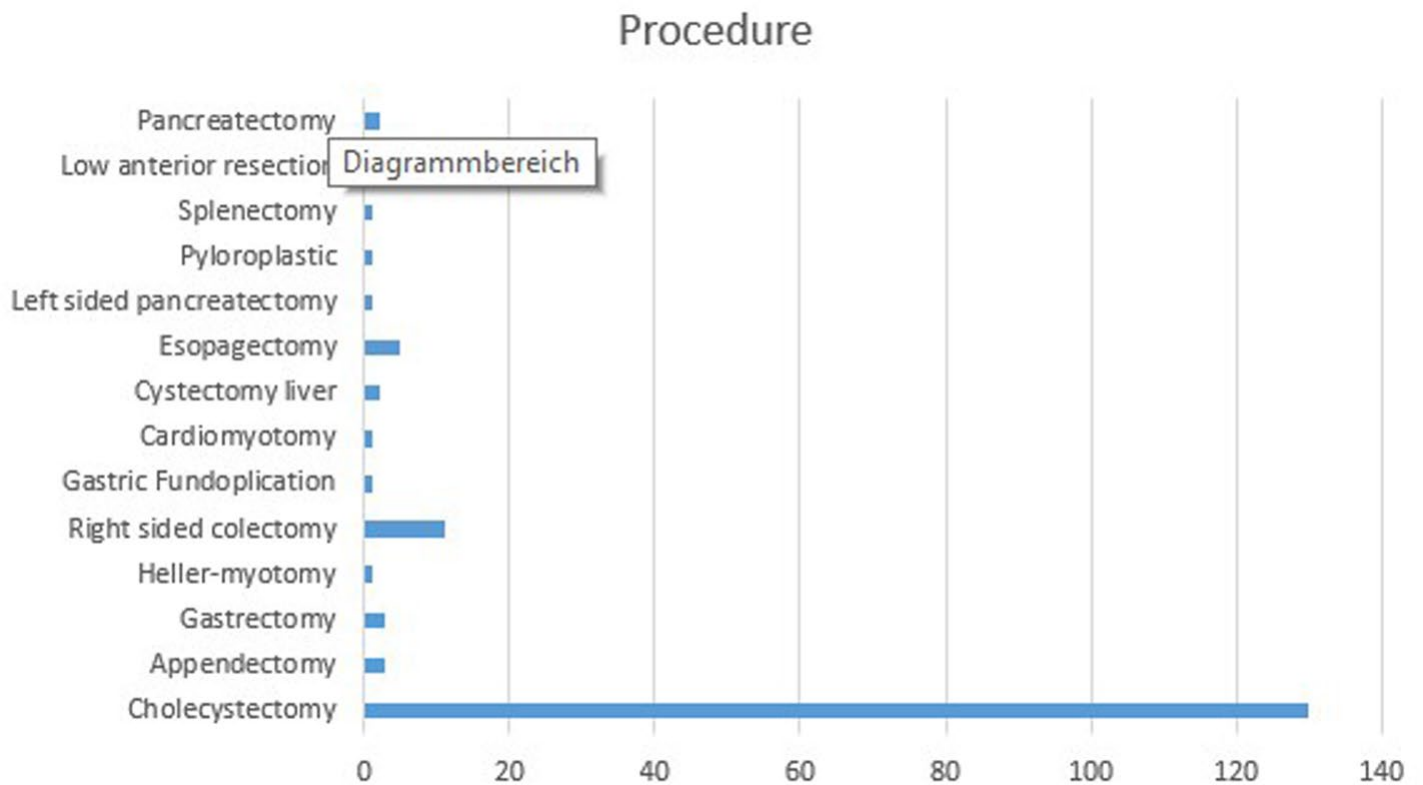
175 robotic assisted operations

175 patients received robotic surgery with the Versius system. 100 female and 75 male patients received surgical treatment. Mean age was 55.6 years (range 16–87 years), the mean Body Mass Index was 25.8 kg/m^2^ (range 17–47 kg/m^2^). To implement the Versius system we firstly performed cholecystectomy (*n* = 130). After the first successfully treated patients, we started with larger operations as right sided colectomy (*n* = 11), low anterior resection (*n* = 10), pulmonary lobectomy (*n* = 13), gastrectomy (*n* = 3), resection of the distal oesophagus (*n* = 5) and two pancreatectomies (Fig. 4). Table 1 shows the intra- and perioperative data.

**Table 1:** Intra- and perioperative data

Procedure	*n*	Median age (years)	Median BMI (kg/m^2^)	Median total operative time (min)	Median Blood loss (ml)	Median Hospital stay (days)	Major Complication (> / = grade 3)
Cholecystectomy	130	53,49	27,85	82	10	3	Secondary haemorrhage (*n* = 1)
		IQR = 30	IQR = 7	IQR = 33	IQR = 33	IQR = 2	
Appendectomy	3	52	23,3	72	200	6	Secondary haemorrhage (*n* = 1)
Gastrectomy	3	56	24,49	370	200	13	None
Heller-myotomy	1	34	26	155	0	6	None
Right sided colectomy	11	69	27,7	178	125	10	Secondary haemorrhage (*n* = 1)
		IQR = 18	IQR = 20,3	IQR = 50,7	IQR = 630		
Gastric Fundoplication	1	46	36	262	50	5	None
Cardiomyotomy	1	81	23,3	104	0	6	None
Cystectomy liver	2	68	17,4	73	10	5	None
Esopagectomy	5	72	25,8	454	600	22	Anastomotic leak (*n* = 1)
Left sided pancreatectomy	1	72	25,4	178	800	13	None
Pyloroplastic	1	67	26,3	172	20	6	None
Splenectomy	1	61	28	185	50	5	None
Low anterior resection	13	63	27,4	214	100	9	None
		IQR = 16	IQR = 16	IQR = 84	IQR = 12	IQR = 6	
Pancreatectomy	2	65,5	27,1	416	250	24	Secondary haemorrhage (*n* = 1)

**Fig. 5:**
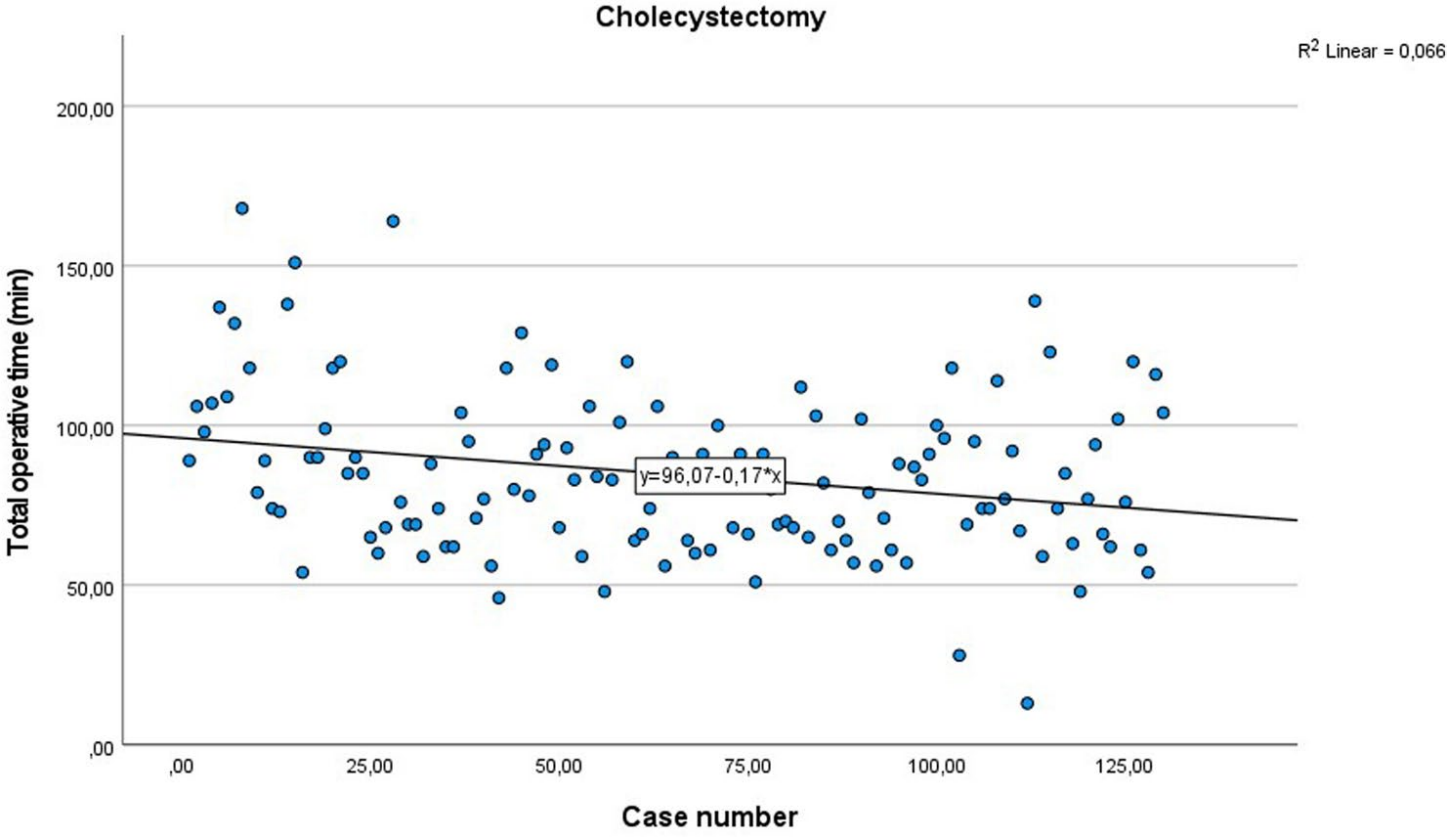
Total operative time addicted to the number of cases

Total hospital stay for minor and major resection was similar to laparoscopic procedure. In most cases, the stay was one or two days less. Furthermore, we saw a fast recovery from surgery. Postoperative paralysis was less than laparoscopic or open surgery. The mean time for return of bowel movement after colorectal surgery was 3.6 days (range 1–6 days). With a rising case number we saw a slow improvement of total operative time (See Fig. 5).

All oncological resections were R0 with a sufficient safety distance to the tumour. With respect to colorectal surgery we payed attention to prepare an intact mesorectal/ mesocolic plane to ensure the quality of the pathological specimen. All colorectal preparations had MERCURY-GRADE 1.

### Complications

Of the 175 patients, four patients had to be revised because of secondary bleeding. One patient underwent surgical treatment for appendectomy. Intraoperative we saw a perityphlitic abscess and diffuse bleeding. The patient had to be revised two days later because of secondary haemorrhage. In one of the cholecystectomies, the patient experienced diffuse liver bleeding that had to be provided after operation. Similar to this case, we revised two other cases (right sided colectomy and pancreatic resection) because of diffuse bleeding (Table 2).

**Table 2:** Complications

Complication	*n*	Treatment
Secondary haemorrhage	4	Operation
Anastomotic leak	1	Endoscopic therapy

One patient developed a leak of the oesophago- jejunal anastomosis followed by a long-term ICU-stay. We did not see any other morbidities. In one case (left sided pancreatectomy), a conversion to open surgery was necessary because of massive collateral veins surrounding the tumour.

## Discussion

To our knowledge, this is the largest cohort of robotic performed operations by using the Versius robotic system in Europe at the time of submission. This series highlights the versatile use of the new Versius robotic system. We believe we have demonstrated the safety and feasibility of performing minor and major surgical interventions with the Versius system. The teams were able to undertake a range of procedures from cholecystectomy, to colorectal surgery, upper-gastrointestinal surgery as well as thoracic surgery. The 3D optics, a much higher optical magnification, wristed instruments, tremor filtration and an independent operator-navigated camera tool are distinct advantages over conventional laparoscopy and can make challenging surgical cases easier to perform. In addition, the open design allows a much better communication between lead surgeon and the entire surgical team.

175 patients received MARS at our departments. Intra- and postoperative data were obtained and under the aspects of safety/complication rate, oncological outcome and operative technique/ease of use evaluated. The oncological outcome as well as the complication rate seem to be similar to conventional laparoscopic or open surgery. Moreover, depending on complexity of case, extent of resection and surgeon’s experience the Versius robotic system seems to be a promising alternative of minimal invasive surgery. The low rate of morbidity and the comfortable posture during surgery are advantages [5, 6]. As expected, during implementation of the system, total operative time was lengthened compared to laparoscopic approach. However, there is a trend of approximation.

The three dimensional, ultra-HD optic with a high magnification level makes it easy to perform surgery in tight spaces like the small pelvis. The oncological outcome and low morbidity are similar to the laparoscopic approach.

We re-operated four patients because of secondary haemorrhage. The bleeding was diffuse. We noticed the difficulty of coagulation firstly during flexure mobilization and opening the Lesser sac as well as performing TME. To optimize the currently limited instrumentation, another coagulation device would be desirable. For intraoperative dissection and coagulation, a bipolar Maryland and a diathermy hook are available. This poses a challenge for performing MARS in patients with a considerable intra-abdominal fat and, furthermore, this is an additional point why the operative time is lengthened [6]. Hence, the effort to develop a new coagulation- device is high. We can expect a new coagulation device until the end of 2022.

Especially under the aspect of hybrid- technique and the fact that the possibility of changing the intraabdominal sight/quadrant during the robotic part is limited, port placement is one of the crucial points performing robotic surgery. Therefore, during the 3.5 day cadaver training we trained and optimized the port positions for our operations and developed a standard to optimize operative procedure [6, 9, 10].

For all positive characteristics of this robotic system, there are limitations that have to be mentioned finally. Beside the need of a new coagulation device, the focal point is always to one space. The opportunity of changing the space e.g. from the upper abdomen to the lower is not given. Hence, hybrid- operations with a laparoscopic part are necessary. Until now, comparisons with other robotic systems do not exist but seem to be essential.

## Conclusions

In our opinion, the Versius Surgical System® seems to be a good, promising system and a safe alternative to other robotic systems, although any comparison is still missing. The open design enabling a better communication between console surgeon and bedside- assistant as well as the mobile bedside units are very interesting and allow more flexibility. Nevertheless, there are limitations of the system that require a direct comparison with other robotic systems as well as continuous advancement.
