# Correction: Clinical implementation of the Versius robotic surgical system in visceral surgery-A single centre experience and review of the first 175 patients

**DOI:** 10.1007/s00464-025-11646-z

**Published:** 2025-03-12

**Authors:** Stefan Wehrmann, Kristin Tischendorf, Torsten Mehlhorn, Annelie Lorenz, Michael Gündel, Hagen Rudolph, Lutz Mirow

**Affiliations:** Department of General and Visceral Surgery, Klinikum Chemnitz gGmbH, Medical Campus of the Technische Universität Dresden, Chemnitz, Germany

**Correction to: Surgical Endoscopy (2022) 37:528–534** 10.1007/s00464-022-09526-x

The original online version of this article was revised to add a notice indicating the authors did not receive direct support from any organization for the submitted work. The Disclosures notice was also emended as follows:

Prof. Lutz Mirow received an expense allowance from CMR Surgical as part of his work as a preceptor. He has supported hospitals that have newly purchased Versisus from CMR in implementing the system. Prof. Lutz Mirow was presented technical innovations in live and online sessions for medical assessment. This consultancy work was compensated by CMR Surgical.

A formal contract exists between Klinikum Chemnitz gGmbH and CMR Surgical. This agreement encompasses the acquisition, maintenance, and upkeep of the Versius system. Furthermore, CMR Surgical has compensated Klinikum Chemnitz gGmbH for hosting doctors from Germany and Europe to observe the Versius system in clinical operation during its first year after installation. Although this institutional relationship does not directly influence the research funding, it is disclosed for the sake of transparency.

The authors Dr. Stefan Wehrmann, Dr. Kristin Tischendorf, Torsten Mehlhorn, Annelie Lorenz, Dr. Michael Gündel, Dr. Hagen Rudolph declared they have no competing interests.

The original article has been corrected.

